# Denervation-Related Neuromuscular Junction Changes: From Degeneration to Regeneration

**DOI:** 10.3389/fnmol.2021.810919

**Published:** 2022-02-24

**Authors:** Xinying Huang, Junjian Jiang, Jianguang Xu

**Affiliations:** ^1^Department of Hand Surgery, Huashan Hospital, Fudan University, Shanghai, China; ^2^Shanghai Medical College, Fudan University, Shanghai, China; ^3^Key Laboratory of Hand Reconstruction, Ministry of Health, Shanghai, China; ^4^Shanghai Key Laboratory of Peripheral Nerve and Microsurgery, Shanghai, China

**Keywords:** neuromuscular junction, denervation, reinnervation, clinical therapy, acetylcholine receptors (AchRs)

## Abstract

Neuromuscular junctions (NMJs) are the key interface between terminal nerves and targeted muscle, which undergo degeneration during denervation periods. Denervation-related NMJs changes limits the recovery level of nerve repair strategies. Insights into mechanisms behind neuromuscular junction degeneration and regeneration, following denervation and reinnervation, are of clinical value. Developing some therapies to maintain or protect structures and functions of NMJs may contribute to a better prognosis. Here, we reviewed previous studies of NMJs focusing on the morphological, functional, and molecular changes after denervation, and if those changes can be reversed after reinnervation. Also, we reviewed about the present probable strategies that have been applied clinically or could still be studied in targeting the neuromuscular junction protection or regeneration improvement.

## Introduction

Neuromuscular junctions (NMJs) are the structural and functional interface between terminal of peripheral nerves and their target muscle, which transfers nerve impulse to muscle contraction. The basic composition of NMJ can be divided into three parts: pre-synaptic area, cleft, and post-synaptic area. The most important medium existing in this structure that switch electrical signal to chemical signal should be acetylcholine (Ach), which is released from the pre-synaptic membrane, combined to acetylcholine receptors (AchRs) on the post-synaptic membrane, and, finally, is retrieved to pre-synaptic area. Typical and mature shape of NMJ is called “pretzel-shape” (Koppel et al., 2019). A number of NMJ gathered in a specific area and shaped to form the motor endplates (MEPs). They arrange into an “M”-like patterned lamella clusters in the muscle tissues (Yin et al., 2019).

The maturation process of NMJ is still ongoing in postnatal period. This includes the rearrangement of nerve terminals, subtype shift of AchRs, and so on. Modifications occur inside and outside the cells in NMJ, and peri-synaptic components also play roles during the process such as the Schwann cells (SCs) (Santosa et al., 2018; Chan et al., 2020; Pęziński et al., 2020).

Denervation can be the outcome of many pathological conditions, such as injury, aging, and cancer cachexia. In these conditions, dysfunction and atrophy of the muscle tissue can be the most common and severe consequence, and degeneration of NMJ may be the key point during these processes. So, it is important to find out the changes of NMJ after denervation in addition to when and how these changes happen. Furthermore, if reinnervation can reverse these changes or not is crucial for determining the targeted therapies in those clinical conditions.

Most of the present studies focusing on this topic are animal experiments. There are several types of animal models which can be mainly divided into chemical, such as botox-induced models, and mechanical denervation like sciatic or other neurectomy (Deng et al., 2021). For studies caring about not only the denervation but also the reinnervation, neurectomy models are used more often for second-stage nerve repair surgery.

## Morphological and Functional Changes of Neuromuscular Junction After Denervation

### Acetylcholine Receptors Density and Motor Endplates Distribution Change

Hartzell and Fambrough (1972) and Fambrough (1974) discussed a lot about AchRs density and sensitivity changes after denervation in rat models and found that intrajunctional AchRs decreased while extrajunctional AchRs increased within 14 days after denervation. Similar results were found in cat models in the study of Steinbach (1981).

**FIGURE 1:**
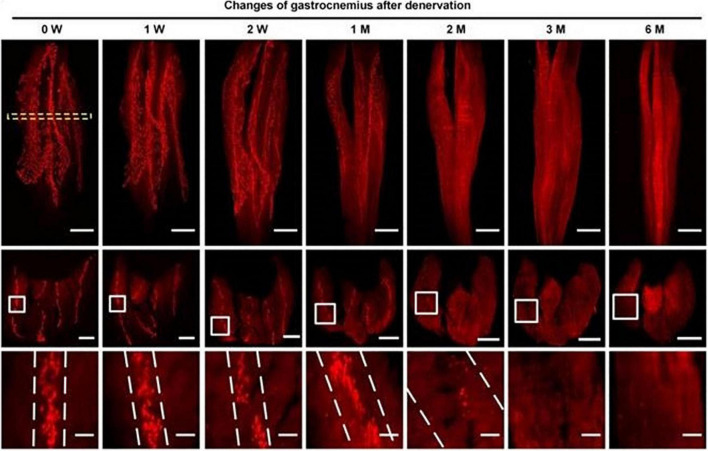
Spatial conformation of acetylcholine receptors (AChRs) stained by α-BTX in motor endplates (MEPs) at different time points. The mean width of the lamella clusters was increased after denervation (Yin et al., 2019).

With the aids of progressive tissue optical clearing technology, Yin et al. (2019) used a 3D imaging technology to observe the spatial distribution of MEPs and its change after denervation in mice models. They found that the mean width of the lamella clusters of MEPs was increased at 2 months after denervation, with a significant decrease in the mean volume of single MEP, which meant that MEPs were disintegrated and were fragmented along the lamella clusters after denervation until they completely disappeared (Figure 1). Other than the findings from animal models, a study of Gupta et al. (2020) presented results from a human body, in which, there were relative absence of normal-appearing MEPs, fragmentation and dispersion of AChRs, and an apparent shift toward plaque-like endplate morphology within 1 year after denervation. Interestingly, after long-term denervation, morphologically preserved MEPs still persisted in muscles that had been denervated for more than 3 years, which means the degeneration process goes more slowly and gradually in human body.

### Higher Turnover Rate of Acetylcholine Receptors and Functional Instability of Neuromuscular Junction

Other than morphological changes, half-time of AchRs decreased, which means a higher turnover rate in short-term after a denervation is caused by surgery (Pumplin and Fambrough, 1982) or toxin (Fumagalli et al., 1990). Therefore, denervation could functionally decrease the stability of NMJs. This kind of higher turnover rate could reduce the functional maturation of AchRs and lead to a metabolic instability of both intra- and extrasynaptic AchRs.

However, studies (Brenner and Rudin, 1989; Rotzler and Brenner, 1990) found that it is the reduced muscle contraction that reduced the half-time of AchRs, but not denervation itself, since direct muscle stimulation protected the stability of NMJs in both innervated and denervated groups.

Hence, we can conclude that after denervation, NMJs in both animals and human bodies have obvious morphological and functional changes. For individual synapses, AChRs dispersed, fragmented, and their turnover increased. Also, the whole arrangement of MEPs changed as they disintegrated. Beyond these changes that happened on NMJ after denervation, it is also important whether there are irreversible molecular changes behind morphological changes and when irreversible changes could happen. In addition, previous studies indicated that NMJs in animal models and human beings have obvious differences, such as the time sequence of these changes, which should be highlighted since they do affect the reference value of many research results from different models, and influence the clinical decision-making for clinicians.

## Molecular Mechanisms Behind Neuromuscular Junction Degeneration After Denervation

Several signaling are involved in the processes of NMJ formation, maturation, and maintenance. Among all the molecular mechanisms, Agrin-LRP4P-MuSK signaling, DOK7 signaling, and Wnt pathways are classic pathways with strong evidence in formation and maintenance of NMJ, while Ach-CDK5-Calpain has more connections with dispersion of AChRs and degeneration of NMJ. Previous reviews (Gonzalez-Freire et al., 2014; Rodríguez Cruz et al., 2020) have systematically discussed about different molecules participating in different physiological and pathological processes, such as aging and some inherited disorders. However, whether these signaling pathways are activated following denervation, can be still confused.

### Mechanism Involved in Changes of Intrinsic Components in Neuromuscular Junctions

**FIGURE 2:**
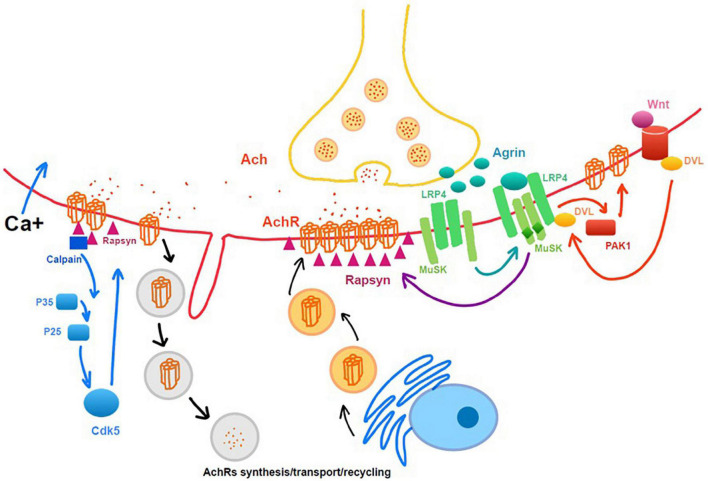
Intrinsic molecular changes related to neuromuscular junction (NMJ) maintenance and degeneration after denervation. Rapsyn, as a cytoplasmic anchoring protein rapsyn, plays a role in stabilizing the AchRs clusters.

Chao et al. (2013) found that Agrin-LRP4P-MuSK signaling help the maintenance of NMJ following a nerve injury by using the MMP3 knockout mice model, which preserved the neural agrin and helped the clustering of AchRs. In study of Kurimoto et al. (2015), they demonstrated that Wnt3a was upregulated, and β-catenin was activated following a traumatic nerve injury, which induced NMJ dispersion and destabilization (Figure 2). The Ca^2+^-p35-p25-dependent proteolysis can be another important muscle cell degradative system that is activated by denervation. It leads to the dispersal of AChR clusters (Machado et al., 2019).

Also, Lok et al. (2007) demonstrated that Gβ subunits, which are located at the NMJ and negatively regulate NRG-mediated MEK-ERK activation, were upregulated after a nerve injury. This played a negative role in regeneration and differentiation of the post-synaptic muscle cells at NMJ. Grosheva et al. (2016) and Rink et al. (2020) found an elevated expression of FGF2 and a reduced expression of BDNF, following a facial nerve injury as two kinds of neurotrophic expression induced a striking reduction in poly-innervated endplates in the NMJs and disturbed a complete recovery.

### Perisynaptic Components Participation in Neuromuscular Junction Remodeling

**FIGURE 3:**
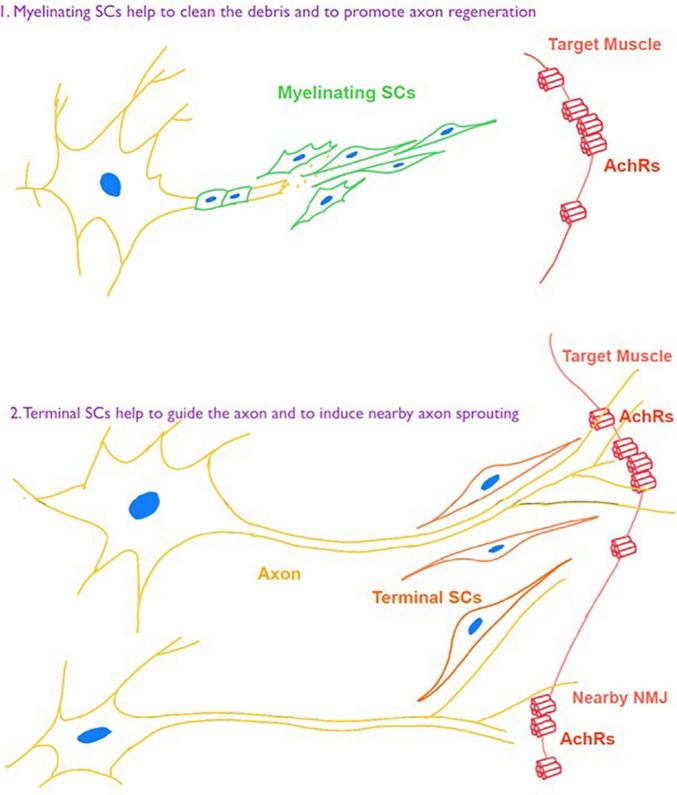
Schwann cell (SC) responses toward denervation for nerve repair and NMJ regeneration.

Besides intrinsic changes of NMJ, perisynaptic cells participate in the process of NMJ remodeling following denervation. Among them, perisynaptic Schwann cells play essential roles in the development and maintenance of NMJ. Also, their ability is related to the time of denervation. In models of rodents, SCs showed an impaired ability of supporting regeneration of axon and NMJ after denervation for more than 4–8 weeks (Sulaiman and Gordon, 2000), and the time can be longer in human body (Ebenezer et al., 2007). Jablonka-Shariff et al. (2020) demonstrated that a GPCR Gpr126 was critically important for not only myelinating SCs to the nerve regeneration, but also in terminal SCs for NMJ maintenance after nerve injury. Also, Lu et al. (2020) demonstrated beneficial contributions of a macrophage-mediated response of NMJs through the induction of terminal SCs response (Figure 3).

All in all, molecular changes of intra and extra components of NMJs happen following denervation. Some of them play a protective role for NMJ maintenance and regeneration, while some of them may negatively affect the morphology and function of NMJs which could then limit the potentiality of function recovery after reinnervation.

## Regeneration of Neuromuscular Junction After Reinnervation and Its Limitation

Functional recovery is a core for evaluating the effect of therapies targeting denervation such as nerve transfer or grafting surgery. The connection between re-dominating nerves and their target muscle can be essential for recovery level, which depends on maintenance and regeneration of NMJs. The reinnervation process, including nerve regeneration, MEPs reunion, and NMJs, reform.

Since MEPs arranged in the lamella cluster and disperse along the similar line following denervation, Yin et al. (2019) investigated if NMJs could regain the original pattern and found that anatomical organization of MEPs is apparently “regained” after reinnervation. However, in the study of Li et al. (2022), they showed that a delayed repair (3 months) could restore the spatial distribution of MEPs, but adversely affect the homogeneity of MEPs and lamellar MEP clusters in mice. Similar results from Vannucci et al. (2019) demonstrated that despite high percentage of NMJs reinnervation, morphological changes, including polyinnervation, axonal sprouting, and endplate fragmentation, occurred with reinnervation.

During the process of reinnervation, there is an important phenomenon called “NMJ-polyinnervation” because of nerve sprouting. Going into details, Rich and Lichtman (1989) found that during the first week of reinnervation, many of the endplate sites are innervated by more than one axon, while within 2 weeks after reinnervation, nerve terminal staining shows that most of the sprouts have regressed. However, this condition of polyinnervation reduced the effectiveness of signaling transduction, which resulted in poor recovery of functional NMJ (Brown et al., 1981). Neurotrophic factors, including CNTF, BDNF, GDNF families, and so on, are related to this pathological process (Sendtner, 1998; Hernandez-Morato et al., 2014; Raimondo et al., 2019; Rink et al., 2020), and their antibodies application could reduce the polyinnervation and improve NMJ recovery.

The NRG-1/ErbB signaling is an important pathway that participates in the stability maintenance of AchRs by modulating Agrin-induced AchR clustering (Schmidt et al., 2011; Ngo et al., 2012). Morano et al. (2018) demonstrated that the regulation of NRG-1/ErbB system correlated to the reinnervation phase, especially ErbB4, has tighter relationship with the NMJs. Muscle activity also has an impact on NMJ regeneration during reinnervation process. After denervation, several signaling systems are activated, and this eventually leads to muscle atrophy and fibrosis in long-term denervation. Interestingly, Macpherson et al. (2015) found that among pathways activated in denervated muscle, Gdf5 and Myog systems also regulated reinnervation process. They demonstrated that when Gdf5 and Myog were inhibited by Dach2-Hdac9, muscle reinnervation was also impaired, which indicated the interaction between the muscle and the nerve activity.

As discussed before, perisynaptic cells are not only involved after denervation. They also participate in NMJs’ regeneration following reinnervation. The most important cells in the process might be SCs since they could guide the growth of regenerative nerve and remodel NMJs (Kang et al., 2019). Furthermore, ECM components cannot be ignored since Brayman et al. (2021) identified the roles for the ECM molecules, Hevin and SPARC for NMJ repair, as they were be upregulated by muscles during reinnervation to orchestrate the fidelity of NMJ repair, at the expense of its rate of reinnervation.

## Neuromuscular Junction Protection Strategies and Their Clinical Value

Neuromuscular junctions degenerate after denervation, and the time between denervation and successful reinnervation could be the most important factor that limit their regeneration (Scheib and Höke, 2013). Clinically, immediate therapies or surgeries for patients are sometimes not available. So, it is important to find out some methods to protect NMJs during the interval period. Besides the earlier nerve repair and acceleration of nerve regeneration, strategies for NMJ protection can target to synapse integration, SCs to function maintenance, and micro-environment modulation.

### Synapse Integration Maintenance

For synapse integration maintenance, there are several strategies that have been discussed. Electrical stimulation has beneficial effects for AchRs clustering for NMJ formation (Lozano et al., 2016) which has been widely applied for patients with peripheral nerve injury. To reduce the inflammation response, McAvoy et al. (2019) developed a microelectrode array which successfully stabilize the AchRs during long-term denervation. As discussed before, Agrin-LRP4P-MuSK signaling can be important for the maintenance of NMJs, therapy-targeting MuSK, which is thought to be of great clinical value, has been attempted to preserve NMJ (Cantor et al., 2018; Feng et al., 2021). However, most studies focusing on these therapeutic medicines are still limited in the animal trials stage.

Furthermore, for those cases in which original dominate nerve repair cannot be achieved, compensatory nerve sprouting can also be a potential therapeutic strategy for muscle function preservation (Marshall and Farah, 2021).

### Schwann Cells Function Recovery

Schwann cells play an irreplaceable role during NMJ regeneration since they could guide axons to achieve the original NMJs sites and remyelinate them (Gordon, 2020; Nocera and Jacob, 2020). The GH-IGF-1 system has been proven to have the potential to improve outcomes after the nerve injury (Tuffaha et al., 2016; Lopez et al., 2019; Slavin et al., 2021). Also, it has been demonstrated that during the regeneration period, GH and IGF-1 support SCs myelination and inhibit SCs apoptosis. What is gratifying is that some drugs in this area have been approved by the Food and Drug Administration (FDA), such as Tesamorelin, for other diseases rather than denervation (Traynor, 2010). Clinical trials are promising and are to be carried out for peripheral nerve injury.

Emerging therapeutic strategies, including human muscle-derived stem/progenitor cell (hMDSC) transplantation, has been studied by Lavasani et al. (2014), and they found that hMDSCs differentiated into myelin-producing SCs and helped NMJs reorganization, which promised a potential clinical application.

### Supporting Environment Regulation

Supporting environment can also be essential for NMJ regeneration, including interstitial cells and components, cytokines, inflammatory cells, and so on. As discussed before, Neuregulin (NRG) is an important neurotrophic factor that improved the NMJ regeneration in animal studies and found that NRG-1 works for NMJs protection after denervation (Fricker et al., 2011; Mancuso et al., 2016).

Another component in the environment around NMJs can be the infiltrating inflammation cell, such as mast cells (Trias et al., 2017) and macrophages (Mokarram et al., 2012), some of them infiltrated around the denervated NMJ, Masitinib, which normalize their infiltration, decreased NMJ degeneration, which has already undergone clinical trials for ALS patients. Furthermore, muscle satellite cells (MuSCs) are vital for muscle regeneration following any type of muscle injury including denervation (Wong et al., 2021). Also, impaired function of MuSCs lead to poor regeneration of NMJs (Liu et al., 2015, 2017).

## Conclusion

All in all, along with the denervation, NMJs changed and degenerated gradually to an irreversible level. Functional recovery would be restricted after the clinical nerve repair therapy because of these irreversible degenerations. While in clinical work, there are several situations in which the interval time before reinnervation strategies cannot be avoided. So, it could be of great clinical value to develop some medicines or other types of therapies [e.g., exercise (Nishimune et al., 2014)] to maintain the integrity and functions of NMJs for better prognosis. Furthermore, it should be highlighted that our review mainly focused on previous studies that targeted adults. Similar topics in children should be distinguished due to the differences between adult and pediatric treatment. For example, NMJs still undergo maturation period during the neonatal period such as the AchR γ-ε subunits switch (Missias et al., 1996).

## Author Contributions

JJ and JX designed the topic. XH collected the reference and drafted the manuscript. All authors made contributions to the conception and design of the study, acquisition of data, analysis, and interpretation of data, drafting the article or revising it, and gave final approval of the submitted version.

## Conflict of Interest

The authors declare that the research was conducted in the absence of any commercial or financial relationships that could be construed as a potential conflict of interest.

## Publisher’s Note

All claims expressed in this article are solely those of the authors and do not necessarily represent those of their affiliated organizations, or those of the publisher, the editors and the reviewers. Any product that may be evaluated in this article, or claim that may be made by its manufacturer, is not guaranteed or endorsed by the publisher.

## Abbreviations

NMJ: neuromuscular junction
Ach: acetylcholine
AchRs: acetylcholine receptors
MEPs: motor endplates
SCs: Schwann cells
NRG: Neuregulin.
